# Differential phylogenetic expansions in BAHD acyltransferases across five angiosperm taxa and evidence of divergent expression among *Populus *paralogues

**DOI:** 10.1186/1471-2164-12-236

**Published:** 2011-05-12

**Authors:** Lindsey K Tuominen, Virgil E Johnson, Chung-Jui Tsai

**Affiliations:** 1 Warnell School of Forestry and Natural Resources, University of Georgia, Athens, GA 30602-2152, USA; 2 Department of Genetics, University of Georgia, Athens, GA 30602-7223, USA

## Abstract

**Background:**

BAHD acyltransferases are involved in the synthesis and elaboration of a wide variety of secondary metabolites. Previous research has shown that characterized proteins from this family fall broadly into five major clades and contain two conserved protein motifs. Here, we aimed to expand the understanding of BAHD acyltransferase diversity in plants through genome-wide analysis across five angiosperm taxa. We focus particularly on *Populus*, a woody perennial known to produce an abundance of secondary metabolites.

**Results:**

Phylogenetic analysis of putative BAHD acyltransferase sequences from *Arabidopsis*, *Medicago*, *Oryza*, *Populus*, and *Vitis*, along with previously characterized proteins, supported a refined grouping of eight major clades for this family. Taxon-specific clustering of many BAHD family members appears pervasive in angiosperms. We identified two new multi-clade motifs and numerous clade-specific motifs, several of which have been implicated in BAHD function by previous structural and mutagenesis research. Gene duplication and expression data for *Populus*-dominated subclades revealed that several paralogous BAHD members in this genus might have already undergone functional divergence.

**Conclusions:**

Differential, taxon-specific BAHD family expansion via gene duplication could be an evolutionary process contributing to metabolic diversity across plant taxa. Gene expression divergence among some *Populus *paralogues highlights possible distinctions between their biochemical and physiological functions. The newly discovered motifs, especially the clade-specific motifs, should facilitate future functional study of substrate and donor specificity among BAHD enzymes.

## Background

BAHD acyltransferases make up a large family of enzymes responsible for acyl-CoA dependent acylation of secondary metabolites, typically resulting in the formation of esters and amides. In a foundational paper, St. Pierre & De Luca [1] named the family after the first four characterized members (**B**EAT or benzylalcohol *O*-acetyltransferase from *Clarkia breweri*; **A**HCTs or anthocyanin *O*-hydroxycinnamoyltransferases from *Petunia*, *Senecio*, *Gentiana*, *Perilla*, and *Lavandula*; **H**CBT or anthranilate *N*-hydroxycinnamoyl/benzoyltransferase from *Dianthus caryophyllus*; **D**AT or deacetylvindoline 4-*O*-acetyltransferase from *Catharanthus roseus*). Currently, the BAHD family encompasses over sixty biochemically characterized members in plant taxa ranging from gymnosperms to monocots to legumes. Previous work has shown that these enzymes may be involved in synthesis or modification of such diverse metabolites as alkaloids, terpenoids and phenolics, with ecophysiological roles in minimizing cuticular water loss, defending against herbivory, and attracting pollinators (reviewed in [2]).

The BAHD family has been previously organized into five major phylogenetic clades, using 46 biochemically or genetically characterized members [2]. This classification revealed both clade-specific and clade-independent biochemical activities among family members. For example, benzoyl-CoA donor utilization so far appears to be limited to Clade V, while hydroxycinnamoyl-CoA has been reported as a donor for members in multiple clades [2]. Substrate specificity typically varies among clades, and sometimes within clade as well. For example, Clade I members act mainly upon flavonoids, while Clade V members utilize substrates ranging from terpenoids to medium-chain alcohols to quinic acid, in association with major phylogenetic branches within this clade [2]. Similar diversity of function was also noted for Clade III members, which are involved in formation of alkaloids, esters, and flavonoids, but functional association was less clear due to the smaller size of subclades in this branch. This highlights both the diversity of the BAHD family and the potential challenge of phylogeny-based functional inference with limited sequence and/or species representation.

Most functionally characterized BAHD acyltransferases share two conserved motifs, HXXXD and DFGWG [2]. The conservation of these motifs has facilitated *in silico *identification of BAHD acyltransferases from available genome sequences [3,4]. The HXXXD motif is also found in other thioester CoA-utilizing acyltransferase families [1] and is absolutely conserved among BAHD acyltransferases. Its importance for catalysis was first established by site-directed mutagenesis [5,6]. Crystallographic analysis of the chrysanthemum (*Dendranthema *× *morifolium*) malonyltransferase Dm3MaT3 provided the structural basis for the catalytic role of the His residue in malonyl-CoA binding [7]. The importance of the DFGWG motif, which is highly but not absolutely conserved, for enzyme activity was first shown in a *Salvia *malonyltransferase [5] and a *Rauvolfia *vinorine synthase [6] based on mutagenesis studies of the Asp residue. However, structural analysis of Dm3MaT3 suggested that this Asp residue most likely plays a structural, rather than catalytic, role in enzyme function [7]. Coupling the structural analysis with mutagenesis studies of two other malonyltransferases from the same species also revealed a greater structural diversity of acyl acceptor binding sites relative to the acyl-CoA donor binding sites [7]. This is consistent with the known broad range of acceptor molecules and relatively narrow range of acyl-donors utilized by different BAHD acyltransferases [2].

Despite the prevalence of BAHD acyltransferases in plants, cross-genome analysis of this family is lacking. Genome-wide analyses of this family have recently been reported for *Arabidopsis *and *Populus*[3,4], but only in a single-taxon context. We sought to explore BAHD acyltransferase diversity from an evolutionary perspective, with a primary focus in *Populus *due to its ability to synthesize a broad array of secondary metabolites. The most abundant of these metabolites are the phenylpropanoid-derived non-structural phenolics known to play significant roles in biotic and abiotic stress responses in this genus [8,9]. The diversity of *Populus *phenylpropanoids (e.g., hydroxycinnamate derivatives, flavonoids, condensed tannins and salicylate-containing phenolic glycosides) can be attributed in large part to side-chain modifications, such as glycosylation, methylation, and acylation [8]. We therefore used a phylogenomic approach to develop an updated phylogeny of the *Populus *BAHD acyltransferase family in reference to four other angiosperm taxa. Together with gene duplication and expression analyses, our data suggest that lineage-specific gene duplication is a key process in BAHD family evolution. The results are consistent with a role of the BAHD acyltransferases in diversifying the secondary metabolite repertoire in plants.

## Results

### *Populus *Has More BAHD Acyltransferase Genes Than *Arabidopsis*, *Medicago*, *Oryza*, and *Vitis*

BLASTP searches against the JGI *Populus trichocarpa *genome release v1.1 revealed 149 unique loci with high similarity to biochemically characterized BAHD acyltransferases from a previous review [2]. Manual curation and referencing against the recently released genome v2.0 were conducted to exclude loci lacking a conserved motif (HXXXD or DFGWG), loci that represented redundant, possibly allelic copies, and loci resembling spurious gene models (see Methods). The final list of 100 putative *Populus *BAHD acyltransferases was used for all subsequent analyses and annotation (Additional File 1: SupplementalTable1.xls). In the course of our work, another group also annotated the BAHD family in *Populus*[3] and reported 94 putative gene models. These models correspond to 74 putative BAHD genes on our list, with one model that matched two v2.0 gene models on our list; the 21 remaining models were either redundant or rejected based on our manual curation criteria (Additional File 1). Similar BLAST search and quality control measures were also performed for the genomes of *Arabidopsis*, *Medicago, Oryza*, and *Vitis*, producing final lists of 55, 50, 84, and 52 putative BAHD genes, respectively (Additional File 2: SupplementalTable2.xls). These lists include ten biochemically characterized *Arabidopsis *members and one biochemically characterized *Medicago *member (see Additional File 2 for references).

### Phylogenetic Analysis Supports Eight Major Clades of Plant BAHD Acyltransferases

**Figure 1:**
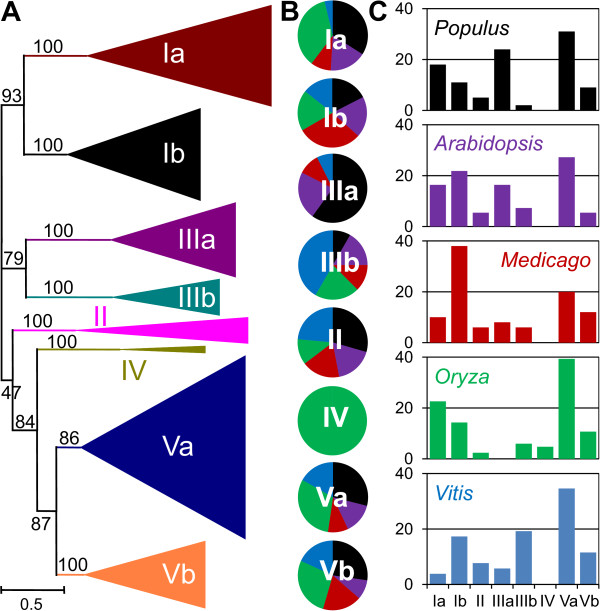
**Phylogeny and Distribution of BAHD Acyltransferases**. A: Protein phylogeny of biochemically characterized BAHD acyltransferases and putative BAHD proteins from *Arabidopsis*, *Medicago*, *Oryza*, *Populus*, and *Vitis *genomes. Phylogeny was constructed using maximum likelihood analysis. B: Percentage representation of putative BAHD acyltransferases across the five taxa within each phylogenetic clade. Colors correspond to the plant taxa as listed in C. C: Percentage representation of clade membership for putative BAHD acyltransferases within each plant genome.

Phylogenetic relationships among the BAHD acyltransferases were reconstructed using a maximum-likelihood algorithm, for a collection of 69 biochemically characterized plant BAHD acyltransferases and the putative members from *Populus*, *Arabidopsis*, *Oryza*, *Medicago*, and *Vitis *(Figure 1A). The resulting phylogenetic tree is broadly consistent with that of D'Auria [2], who sorted biochemically characterized BAHD acyltransferases into five major groups. Our expanded analysis suggests that a grouping of eight major clades is now warranted, a finding consistent with previous, single-genome-based, neighbour-joining analyses [3,4,10]. In particular, a strongly-supported clade comprised entirely of BAHD acyltransferases lacking biochemical characterization data was sister to the group of proteins previously designated as Clade I by D'Auria [2]. To maintain consistency, we adopted a similar clade nomenclature, and name the previous and the "new" groups as Clades Ia and Ib, respectively. Clades Ia and Ib correspond respectively to the *Populus *clades Vb+Vc and Va, and to the *Arabidopsis *clades IIb and IIa reported by Yu et al. [3]. Another strongly supported clade containing the *Petunia *acetyl CoA:coniferyl alcohol acetyltransferase (CFAT, [11]) was sister to the group classified by D'Auria as Clade III [2]. We name the previous and the "new" clades as IIIa and IIIb, respectively; these correspond to the *Populus *clades IV and II and *Arabidopsis *clades IV and IIIa in Yu et al. [3]. Members of the former Clade V [2] clustered into two well-supported groups in our analysis, renamed hereafter Clades Va and Vb. These clades correspond to Yu et al.'s clades Ia and Ib for both *Populus *and *Arabidopsis*[3]. Characterized proteins in Clade Va tend to be involved in volatile ester formation, while those in Clade Vb are closely related to hydroxycinnamoyltransferases (HCTs) responsible for the synthesis of chlorogenic acid and monolignols. Our analysis also placed Clade IV basal to Clades Va and Vb, with good support. The remaining sequences clustered into one strongly supported group corresponding to D'Auria's Clade II [2].

The distribution of sequences among the five species varied within each clade (Figure 1B). *Populus *and *Oryza *have the largest number of BAHD members overall, and collectively these made up the majority of Clades Ia, Va, and Vb. *Populus *also predominated in the dicot-specific Clade IIIa, while Clade IV was monocot-specific. Taxon bias was also evident in Clades Ib and IIIb, where *Medicago *and *Vitis*, respectively, were over-represented. When analyzed by species, Clade Va, the largest clade, remained the largest group in all taxa, except in *Medicago *where Clade Ib predominated (Figure 1C). Clades II, IIIb, and IV had the lowest representations overall, consistent with their small overall sizes. The major exception to this pattern was *Vitis*, which showed a relatively higher representation of Clade IIIb, coinciding with a much lower representation of Clade Ia. Other species-biased patterns included high (>20%) representation for Clade IIIa in *Populus*, Clade Ib in *Arabidopsis*, and Clade Ia in *Oryza*.

**Figure 2:**
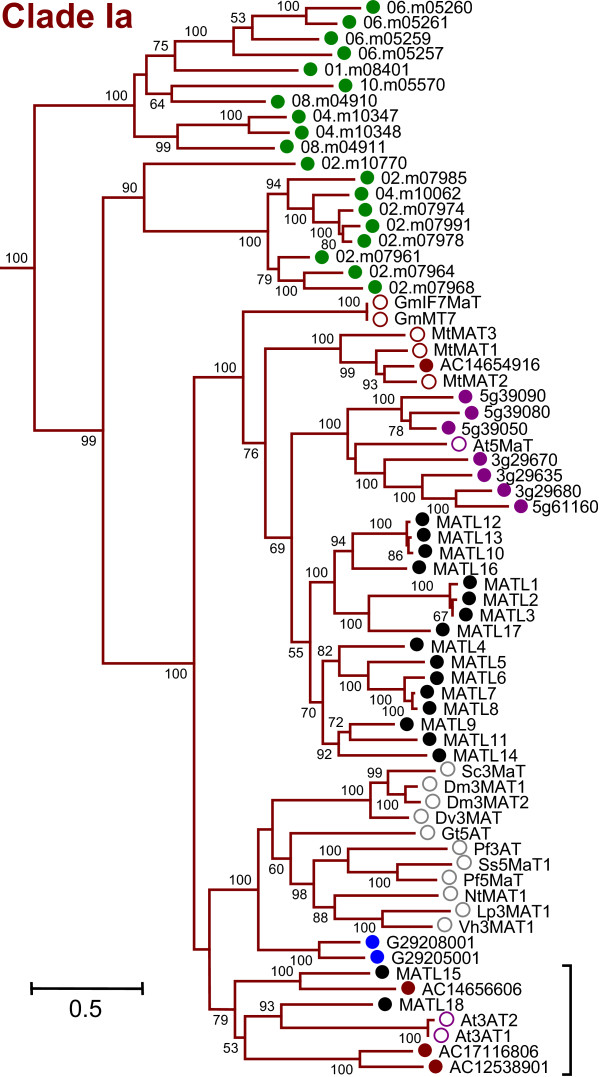
**Phylogenetic Relationship of Clade Ia Members**. Expanded view of all Clade Ia sequences from Figure 1A. Bracket indicates region lacking taxon-specific clustering. Filled circles represent putative BAHD acyltransferases, while open circles represent characterized BAHD proteins. Colors correspond to taxa as listed in Figure 1, with gray circles indicating sequences from plants within the Asterids. *Populus *sequence names are provided in Additional File 1. Loci from the other four genomes have been truncated to accommodate text input limitations (e.g., 1g03495 for At1g03495 of *Arabidopsis*, AC1253891 for AC125389_1 of *Medicago*, 01.m08401 for 12001.m08401 of *Oryza*, G29205001 for GSVIVP00029205001 of *Vitis*). GenBank accession numbers and full names for previously characterized proteins are provided in Additional File 10.

Closer examination of the phylogeny revealed that BAHD sequences from a single taxon tended to cluster together, especially within the larger clades. In Clade Ia, all sequences from the five taxa formed lineage-specific groups with strong bootstrap support, except for one well-supported subgroup (Figure 2, bracket). *Oryza *sequences were basal to all eudicot sequences in this clade. Two strongly-supported subclades consisting of a combined total of sixteen *Populus *sequences comprised another large, but in this case weakly-supported, group, sister to a group of eight *Arabidopsis *sequences, including a malonyl CoA:cyanidin 3,5-diglucoside transferase (At5MaT, [4,10]; Figure 2). Similar, but less dramatic patterns were observed for Clade Ib (Additional File 3: SupplementalFigure1.png). While the two most basal subgroups in this clade did not show strong taxon specificity, the two remaining subgroups each comprised five taxon-specific branches with strong support (Additional File 3). In accordance with its overrepresentation overall in Clade Ib, *Medicago *exhibited substantial taxon-specific expansions within these two branches.

**Figure 3:**
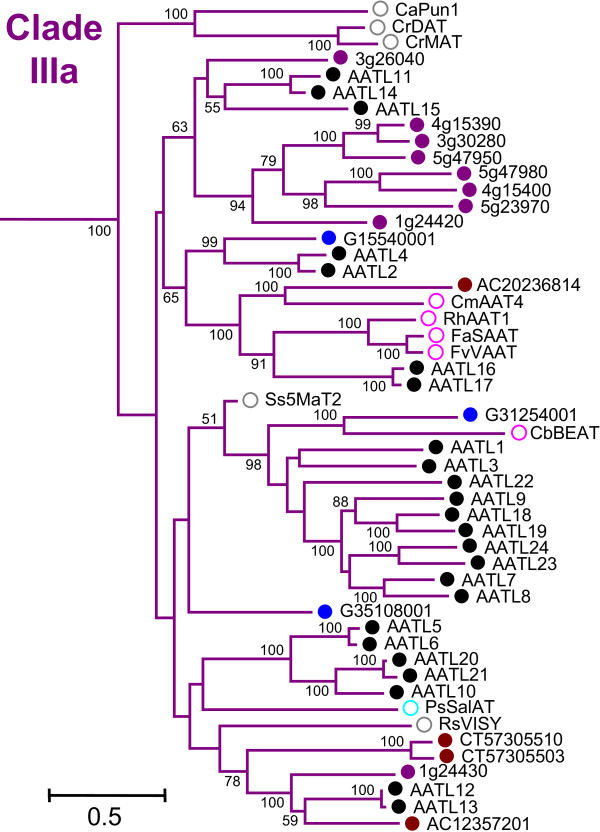
**Phylogenetic Relationship of Clade III Members**. Expanded view of all Clade III sequences from Figure 1A. Colors and symbols are the same as in Figures 1 and 2. In addition, pink circles indicate sequences from plants within the Rosids, while teal circle indicates sequence from a basal eudicot.

Taxon-specific clustering appeared more scattered in Clade IIIa, perhaps because the larger of the two major branches was poorly resolved (Figure 3). Ten *Populus *sequences formed a well-supported subclade together with a *Clarkia breweri *acetyltransferase involved in benzyl acetate formation (CbBEAT, [12]), and with an uncharacterized *Vitis *sequence. A smaller subclade contained five *Populus *sequences, and a third taxon-specific subclade containing seven of the nine *Arabidopsis *sequences in Clade IIIa also had high bootstrap support.

**Figure 4:**
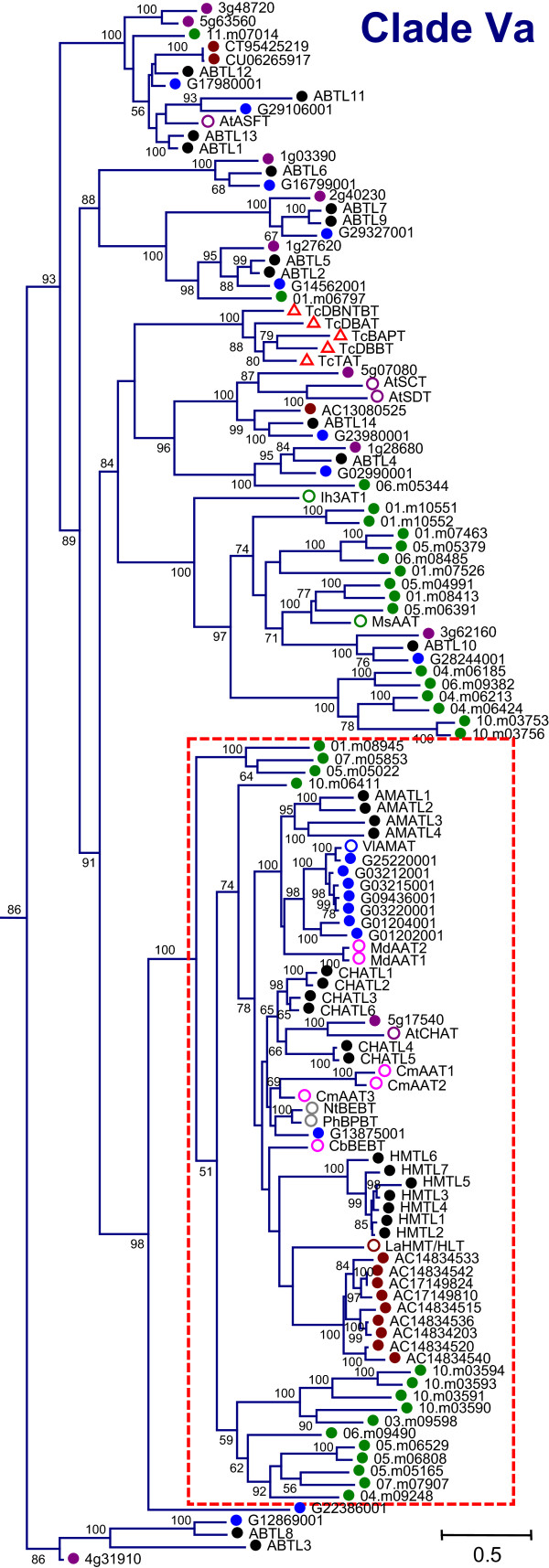
**Phylogenetic Relationship of Clade Va Members**. Expanded view of all Clade Va sequences from Figure 1A. Colors and symbols are the same as in Figures 1-3. In addition, red triangles indicate sequences from gymnosperms. Boxed region indicates a poorly resolved branch based on bootstrap analysis.

As the largest phylogenetic group, Clade Va contained a number of highly-derived branches, some specific to gymnosperms, monocots, or dicots (Figure 4). The largest well-supported branch in this clade contained four taxon-specific clusters of at least seven members (Figure 4, boxed), one each for *Vitis *(eight members), *Populus *(seven), *Medicago *(nine), and *Oryza *(eleven). *Oryza *sequences were over-represented in this clade and fell mainly into two large branches with moderate bootstrap support. One was *Oryza*-specific as mentioned above, and the other contained three eudicot sequences (Figure 4). Taxon-specific clustering was not as evident in Clade Vb, except for a well-supported branch of seven *Oryza *sequences, sister to a group of hydroxycinnamoyltransferases (HCT/HQT) involved in biosynthesis of lignin, chlorogenic acid, and other phytoalexins (Additional File 3).

Clade II lacked species-specific clustering patterns, as members were more evenly distributed among species (Additional File 3). Clade IIIb was relatively small, and exhibited some degree of taxon-specific clustering. The largest such grouping comprised nine *Vitis *sequences, consistent with their overrepresentation in this clade (Additional File 3). A four-member subclade of *Oryza *sequences and a three-member subclade each for *Arabidopsis *and *Medicago *were also evident. Clade IV was the smallest clade and was restricted to monocots, as mentioned previously.

With regard to *Populus*, species-specific expansion was evidenced within Clades Ia, IIIa and Va. Because the *Populus*-specific subgroup in Clade Ia is most closely related to several biochemically characterized malonyltransferases from *Arabidopsis*, *Medicago*, and *Glycine*, we have named members of this clade as malonyltransferase-like (MATLs). The sequences in the *Populus*-specific branch are MATL1-14 and 16-17. We designated all *Populus *sequences in Clade IIIa as alcohol acyltransferase-like (AATLs), after the numerous characterized alcohol acyltransferases within that clade. The *Populus*-specific branch includes AATL1, 3, 7-9, 18-19, and 22-24. We refer to the three *Populus *clusters within the largest branch of Group Va by three names. First, we named the set of four *Populus *sequences clustering with two *Malus *sequences and a set of *Vitis *sequences, including an anthraniloyl-CoA:methanol acyltransferase from *Vitis labrusca *(VlAMAT, [13]), as AMAT-like (AMATLs). Next, we refer to the six *Populus *proteins most closely related to the *Arabidopsis *acetyl CoA:*cis*-3-hexen-1-ol acetyl transferase [14] as CHAT-like (CHATLs). Finally, the subgroup of seven *Populus *sequences that fell into a poorly-resolved region of Clade Va, most closely to a tigloyl-CoA:(-)-13*α*-hydroxymultiflorine/(+)-13*α*-hydroxylupanine *O*-tigloyltransferase from *Lupinus *(LaHMT/HLT, [15]), were named HMT-like (HMTLs).

### New Family-wide and Clade-Specific Motifs are Present in BAHD Acyltransferases

**Figure 5:**
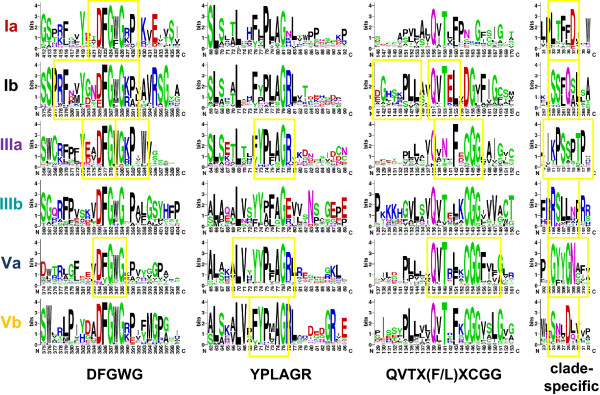
**Conserved Motifs Within Phylogenetic Clades**. WebLogo displays of consensus sequences corresponding to MINER-identified motifs, boxed in yellow. Logos are arranged in rows by phylogenetic clade, named at left, and in columns by motif, labelled at the bottom. The three leftmost columns represent motifs conserved across multiple clades. The rightmost column provides examples of clade-specific motifs; motifs in this column are not aligned relative to one another.

The large number of BAHD genes available from sequenced plant genomes presents an opportunity to expand the analysis of conserved motifs in this family beyond the two known functional domains, HXXXD and DFGWG. We subjected sequences from each clade to motif analysis using MINER v2.0 [16-18]. Clades II and IV were excluded from the analysis due to their small sizes. Using a sequence window of five amino acids and the default z-score threshold, four to nine motifs were predicted for each clade (Figure 5, Additional File 4: SupplementaryFigure2.pdf). MINER identified the DFGWG motif in four of the six tested clades (Ia, Ib, IIIa, and Va). Although it did not meet the MINER threshold, visual inspection revealed high conservation of this motif in Clades IIIb and Vb as well (Figure 5). This supports the validity of our approach towards the identification of conserved motifs. The HXXXD motif escaped detection by MINER, but this was expected since the motif contains a variable core.

Two new motifs were identified with multi-clade conservation. The first motif had a consensus of YPLAGR beginning around position 71-78, and was predicted in Clades IIIa, Va, and Vb. Manual inspection of the other clades identified a similar motif in this region, but with notable variability from the consensus, especially for the two flanking residues (Figure 5). The second motif had a consensus of QVTX(F/L)XCGG around position 136-156 and was predicted in Clades Ib, IIIa, and Va. Manual inspection revealed that QVT was highly conserved in the other three clades, but CGG was poorly conserved in Clades Ia and Ib (Figure 5). Clade-specific motifs were also observed, several of which were located near the N-terminus of the protein: the LTFFD motif from Clade Ia was located at positions 33-37, the IKPSSPTP motif of Clade IIIa at positions 11-18, and SNLDL from Clade Vb at positions 25-29 (Figure 5). Because the N-terminus often contains targeting peptide sequences, we examined the predicted protein subcellular localization patterns by clade using three different prediction programs. However, we found no evidence for a link between the observed clade-specific N-terminal motifs and the predicted subcellular targeting of the BAHD proteins (Additional File 5: SupplementaryFigure3.pdf).

Although Clade II was too small for motif analysis, we note that none of its members would have been accepted using our initial search criteria (both HXXXD and DFGWG present). The two original clade members, ZmGlossy2 and AtCER2, are known to participate in cuticular wax biosynthesis based exclusively on genetic characterization studies [19-21]. In the absence of biochemical data, it remains debatable as to whether Clade II members should be considered true BAHD acyltransferases.

### Multiple Gene Duplication Types Have Contributed To BAHD Family Expansion in the *Populus *Genome

**Figure 6:**
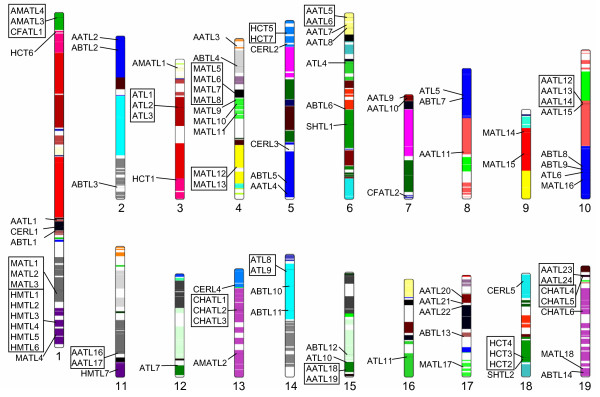
**Locations of Putative *Populus *BAHD Acyltransferases on Linkage Groups**. Homeologous blocks arising from the salicoid genome duplication event are color-coded across the nineteen linkage groups (chromosomes). BAHD acyltransferases in close proximity to one another are boxed for ease of labelling. Note that proximity on a linkage group does not, by itself, indicate a close phylogenetic relationship.

*Populus *has experienced at least two genome-wide duplication events, the salicoid event approximately 60-65 MYA and the older eudicot triplication event, as well as numerous segmental and tandem duplication events [22,23]. We sought to determine whether the various types of gene duplications contributed towards the expansion of the *Populus *BAHD family, especially with regard to *Populus*-specific subclades (HMTLs, CHATLs, and subgroups of MATLs and AATLs). Overall, we found sixty BAHD genes were associated with recent (salicoid or local) duplications (Additional File 6: SupplementalTable3.xls), accounting for more than half of the BAHD acyltransferases in *Populus *(Table 1). This is broadly consistent with previous analysis of chromosomal location of BAHD acyltransferases in *Populus*, which mapped 25 of 58 genes to homeologous chromosome segments or tandem duplication blocks based on the v1.1 genome release [3]. Events were spread approximately evenly across the two duplication types, with a greater number of local (e.g., tandem) duplications overall. Duplications were found in all but the two smallest clades (II and IIIb). Salicoid and local duplications were overrepresented in Clades Ib, Va, and Vb relative to the genome overall. Such duplications impacted every member of Clade Ib (three salicoid pairs, one local pair and one local triplet), all but two genes in the largest subclade of Va (Figure 4, boxed; including two salicoid duplications, three local pairs, one local triplet, and one local quadruplet), and all but one member of Clade Vb (including two local pairs and two salicoid pairs; Figure 6, Additional File 6). For two subclades within the large, poorly resolved region in Clade Va, multiple local duplications appear to have followed genome-wide duplication events in one of the two salicoid paralogues (Figure 6, Additional File 6). The first instance is the relationship between HMTL7 on linkage group (LG) XI and the HMTL1-6 cluster on LG I. The second is the relationship between CHATL6 on LG XIX and the CHATL1-3 triplet on LG XIII.

**Table 1:** Summary of Gene Duplication Events Among *Populus *BAHD Acyltransferases

Clade	Ia	Ib	II	IIIa	IIIb	Va	Vb	Genome
Total genes in clade	18	11	5	24	2	31	9	100
Recent duplication	6 (33%)	11 (100%)	0 (0%)	14 (58%)	0 (0%)	21^a ^(68%)	8 (89%)	60 (60%)
Salicoid duplication	0	6	0	6	0	10	4	26
Local duplication	6^b^	5^c^	0	8	0	13^d^	4	36
Retrotransposon association	7 (39%)	4 (36%)	1 (20%)	4 (17%)	1 (50%)	16 (52%)	2 (22%)	35 (35%)
Both 5' and 3' of gene	5	1	1	2	0	3	1	13
Either 5' or 3' of gene	2	3	0	2	1	13	1	22

^a ^Two members in Clade Va are associated with both salicoid and local duplications (21 = 10+13-2); ^b ^Includes two triplets; ^c ^Includes one triplet; ^d ^Includes one triplet and one quadruplet.

Although Clade IIIa exhibited several duplications, the *Populus*-dominated AATL subclade had just one tandem pair (AATL23 and AATL24). Clade Ia had the lowest rate of duplications among the larger clades, with two local triplets within the *Populus*-dominated MATL subclade (Table 1). The relatively low numbers of local and salicoid duplications in the *Populus*-dominated AATL and MATL subclades raises the possibility that some of these genes might have originated through other mechanisms, such as transposable elements. We therefore searched for the presence of retrotransposons within the two 10-kb windows flanking either side of each *Populus *BAHD gene. We found retrotransposon associations in each clade, covering over one third of the family as a whole, although the majority of associated genes were flanked on only one end (Table 1). Retrotransposon associations were frequently observed for recently duplicated genes (Table 1,  Additional File 6). Retrotransposon associations were overrepresented in Clade Va, noted for all AMATLs and the majority of CHATLs and HMTLs (Table 1, Additional File 6). However, all of these gene models contained at least one intron (Additional File 1), suggesting that retrotransposition is unlikely to be a direct cause of duplication. Retrotransposon associations were underrepresented in Clade IIIa and absent from the AATL *Populu*s-dominated subclade (Table 1,  Additional File 6). Despite its average representation of retrotransposon associations, Clade Ia had the greatest number of genes with retrotransposons flanking both sides (Table 1). Two such genes, MATL12 and 13, formed a strongly supported branch with MATL10. All three are located on LG IV (Figure 6), lack predicted introns (Additional File 1), and share a high degree of nucleotide identity with one another (98%). Although preliminary, our analysis suggests that retrotransposons have contributed to the duplications of some BAHD genes.

### Some Recently Duplicated BAHD Acyltransferases are Differentially Expressed

To investigate expression of *Populus *BAHD genes, we mined a set of nine Affymetrix microarray datasets encompassing five different genotypes and four different tissue types generated in our laboratory [24]. After excluding probes that had consistently low expression across all samples (see Methods) and annotating probes based on the POParray database [25], we obtained expression data for 41 probes corresponding to 48 BAHD genes (some probe sequences match multiple gene targets, and some gene targets are represented by multiple probes). Pairwise correlations of BAHD gene expression across all microarray experiments were computed and the results organized by duplication type (Additional File 7: SupplementalFigure4.pdf). Median Spearman rank correlations were significantly different among the duplication categories according to one-way ANOVA (*p *< 0.001). Not surprisingly, median correlations for gene pairs derived from local or salicoid duplications were significantly higher than for other types of (all possible) gene pairs (Additional File 7).

**Figure 7:**
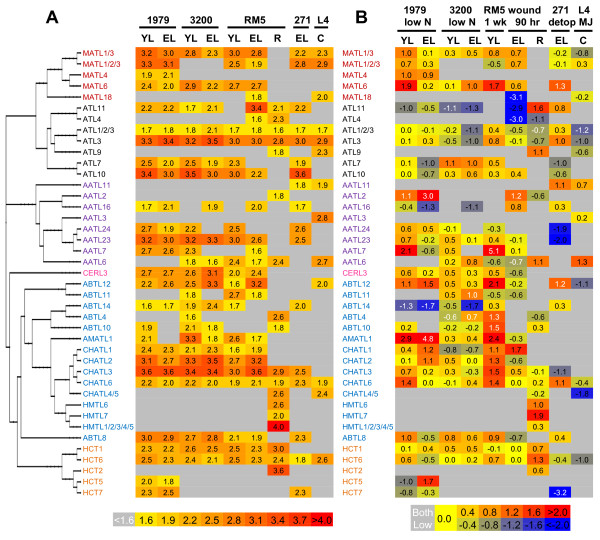
**Expression of BAHD Acyltransferases in *Populus *Tissues, Organized by Phylogenetic Relationship**. A: Expression of BAHD acyltransferase genes across tissues and genotypes. B: Stress responses of BAHD acyltransferase gene expression across tissues and genotypes. Expression data or ratios (stressed vs. control samples) were log-transformed and visualized in heatmaps (see Methods). Genes are organized by phylogenetic relationship and labelled by the clade color in Figure 1. Genotypes analyzed included: *P. fremontii *× *angustifolia *clones 1979, 3200, and RM5, and *P. tremuloides *clones 271 and L4. Tissues analyzed included: young leaf (YL), expanding leaf (EL), root tips (R), and suspension cell cultures (C). Stress treatments included: nitrogen limitation (low N), leaf wounding (wound, sampled either 1 week or 90 hours after wounding), removal of shoot up to leaf plastochron index three (detop, 90 hours after removal), and methyl jasmonate elicitation (MJ) [24]. White text indicates that raw hybridization intensity for either control (for upregulated genes) or stressed treatment (for downregulated genes) samples was below the quantitation limit (see Methods).

When the log-transformed microarray data were visualized as a heatmap, expression across the BAHD family as a whole was biased towards leaves, and we did not observe clear differences in expression patterns among the major clades (Figure 7A). Within the major clades, genotype- and/or tissue-dependent expression patterns were evident. For example, root-specific expression dominated in the HMTL subclade, while the majority of other Clade Va genes showed the more typical leaf-biased expression (Figure 7A). In another case, *HCT1 *and *HCT6 *were relatively uniformly expressed in all three *P. fremontii × angustifolia *hybrid genotypes examined, while *HCT5 *and *HCT7 *were detected only in genotype 1979 (Figure 7A). *HCT2*, on the other hand, was most abundant in roots. Expression patterns diverged for closely related genes in several cases, including genes within the *Populus-*dominated subclades. For example, *MATL4 *was biased towards *P. fremontii *× *angustifolia *genotype 1979 relative to *MATL1-3*, which were more evenly expressed across genotypes and tissues. The *Populus-*dominated AATL subclade includes *AATL3*, which was preferentially expressed in cell suspension cultures, as well as *AATL7*, *23*, and *24*, which exhibited different expression patterns by leaf age and genotype. The *CHATL *cluster includes two members (*CHATL3 *and *6*) that were fairly evenly expressed across sampled tissues, and two (*CHATL1 *and *2*) that were detected only in leaves. The more divergent *CHATL4/5 *were most strongly expressed in non-photosynthetic tissues, yielding an overall pattern that resembled the *HMTLs *more than the other *CHATLs *(Figure 7A).

QPCR was performed to verify the expression patterns of closely related *CHATL *transcripts observed by microarray analysis, using an independent set of *P. tremuloides *tissues (Additional File 8: SupplementalFigure5.pdf). Specific primers were designed to distinguish among the three paralogous pairs with different duplication history (Supplemental Table 2): *CHATL1/2*, *CHATL3/6 *and *CHATL4/5*. *CHATL1/2 *were expressed relatively consistently across all leaf and stem internode tissues sampled, but were lower in root and flower tissues (near or below the corresponding microarray threshold marked by a dotted line in Additional File 8, Panel A). *CHATL3/6 *were most strongly expressed in young leaves and roots, followed by apices and mature leaves, and were much lower in stem and flower tissues. The transcript levels of *CHATL4/5 *were very low overall, with the highest levels detected in roots, similar to the microarray data of *P. fremontii *× *angustifolia*. Overall, the QPCR data were broadly consistent with the microarray results, and support the idea that the three pairs of *CHATL *genes have diverged in their expression patterns despite their high homology.

We next analyzed the microarray data to examine the responses of BAHD gene expression to four different stress treatments, including nitrogen limitation, wounding, detopping, or methyl jasmonate feeding, across several tissues and/or genotypes. Again, no clear overall patterns by clade were observed, and the differential gene expression patterns observed among some paralogous genes described above also held for the stress treatments (Figure 7B). Additional evidence of functional divergence was observed. For example, Clade IIIa member *AATL7 *showed its strongest upregulation in young leaves one week after wounding, while the responses of *AATL23 *and *AATL24 *were most drastically affected via down-regulation in expanding leaves following detopping. The leaf-expressing *CHATL *genes were generally up-regulated by nitrogen stress in *P. fremontii × angustifolia *genotype 1979, except *CHATL6 *in expanding leaves. However, the trend was more variable in genotype 3200 (Figure 7B), despite similar baseline expression of these genes between the two genotypes (Figure 7A). QPCR analysis of the same suite of samples confirmed this general discrepancy between the two genotypes (Additional File 8, Panel B), although the degree of expression changes varied between the two analytical (microarray vs. QPCR) methods. The data hint at differential expression among closely related BAHD genes in response to nitrogen stress between different *Populus *hybrid genotypes. Future investigation would help determine how widespread this pattern is across the BAHD family and a broader range of genotypes in this genus.

## Discussion

### BAHD Family Expansion as a Factor Enabling Metabolic Diversification

Across the five angiosperm genomes investigated here, we observed numerous differential lineage expansions within the BAHD acyltransferase phylogeny. Examination of retained gene copies following duplications in *Populus *revealed that the majority of BAHD genes, at least in this genus, are associated with recent genome-wide as well as local duplication events. An estimated 32% of all v2.0 *Populus *genes (6655 pairs or 13268 unique gene models) were derived from the salicoid duplication event [26]. However, only 26% of the *Populus *BAHD acyltransferases were associated with the salicoid duplication. Tandem or local duplications, on the other hand, accounted for over one-third (36%) of the *Populus *BAHD genes, much higher than the genome average estimated at 16% [23]. It thus appeared that local duplications were over-represented and genome-wide duplications were under-represented in the *Populus *BAHD family relative to the genome average. We speculate that this pattern may be generally applicable to the other angiosperm genomes surveyed in this study. Local duplications might be more likely than polyploidization events to account for the observed taxon-specific expansions of BAHD acyltransferases. This was indeed the case for the *Populus*-dominated HMTL and CHATL subclades, where the majority of the genes were derived from local duplications, and to a much lesser extent, for the MATL subclade. In contrast, only two of the ten members in the *Populus*-dominated subclade among the AATLs were implicated in any duplication event. Preliminary molecular clock analysis suggested that the divergence times among members of the *Populus*-dominated MATL and AATL subclades were similar and predated the salicoid duplication event. This suggests that other duplications, prior to the salicoid duplication event but after the eudicot triplication event, probably contributed to the *Populus*-specific expansion as well.

Previous work has shown that genes involved in stress responses, including secondary metabolic genes, are more likely than average to experience lineage-specific diversification via tandem duplication [27]. When placed in a metabolic pathway context, we suggest that taxon-specific, local duplication-derived expansion of gene (sub)families may be characteristic of enzymes that occupy a terminal or tangential position in a metabolic pathway. Conversely, enzymes with an intermediate position in a core pathway would likely retain a more constant number of gene copies across taxa due to evolutionarily constraint for balanced stoichiometry between enzymes acting within the same pathway. In support of this idea, HCTs known to be involved in intermediate steps of monolignol biosynthesis formed a multi-taxon cluster within Clade Vb, encoded by 1-2 genes in all sequenced genomes. In *Populus*, Clade Vb diversified about equally via salicoid and local duplication events. In contrast, the sister Clade Va exhibited extensive taxon-specific clustering (boxed region, Figure 4); *Populus *genes in the major subclade were associated with more than three times as many local duplications as retained salicoid duplicates. The biochemically characterized enzymes within this branch are all involved in the final step of various volatile ester and alkaloid ester biosynthetic pathways [13-15,28-31].

Taxon-specific phylogenetic expansions have also been observed within the *O*-methyltransferase (OMT) [9,32,33] and glycosyltransferase (GT, especially Group 1) ([34,35], Tsai and Johnson, unpublished) families. Like BAHD acyltransferases, OMTs and GTs form large families, and collectively the three are responsible for the elaboration (acylation, methylation, and glycosylation) of a wide range of secondary metabolites [2,8]. These modifications increase the diversity of natural products, with regard to both their chemical structures and biological activities, and they hold chemotaxonomic value due to their taxon-specificity [36-38]. Studies from the OMT and GT1 families have shown that many of these enzymes possess promiscuous substrate specificity [39,40]. This, coupled with the tendency of taxon-specific diversification of some of the subfamily members, may be a means to afford metabolic plasticity. Consistent with this idea, multifunctional OMTs from *Thalictrum tuberosum *formed a taxon-specific subclade, while those with a limited substrate range formed multi-taxon subclades [33]. In the case of BAHD acyltransferases, numerous biochemically characterized Clade Va members involved in volatile ester biosynthesis have been shown to accept multiple substrates and/or donors, at least *in vitro *[13-15,28,30,31]. Many of these were found in the same subclade where taxon-specific diversification was common (Figure 4, boxed region). These data further support the view that differential lineage expansions of the BAHD family may be linked to taxon-specific metabolic diversification.

### Implications of Divergent Paralogue Expression and Newly Identified Conserved Motifs for BAHD Acyltransferase Function

Given the extent of gene duplications in the *Populus *BAHD family, it was not surprising that many of the closely related members retained similar expression patterns. This, however, was not universal, as we noted for the CHATLs, especially in stressed tissues. While genes with high sequence similarity are likely to have similar biochemical functions, the differential expression patterns observed here suggest that physiological functions might have already diverged for some recent duplicates. Our examination of BAHD acyltransferase expression in *P. fremontii *× *angustifolia *and *P. tremuloides *under various stress conditions builds upon the previous analysis in *P. trichocarpa *by Yu et al. [3]. Overall, these results are broadly consistent in that BAHD expression was generally much stronger in photosynthetic than non-photosynthetic tissues. In the present study, the expression bias towards leaves was generally consistent across genotypes. Relatively little difference was observed between genotypes under control conditions, but differences became evident under stress. Biotic and abiotic stresses are known to influence the relative proportions of secondary metabolites in *Populus *(reviewed by [9,41]), and different genotypes differ in both secondary metabolite diversity and quantities [37,38]. The variety of BAHD expression patterns in stressed tissues, either among recently duplicated genes, between closely related genotypes or during leaf development, is consistent with a role for BAHD genes in regulating secondary metabolite accumulation and diversity in *Populus*.

The two known conserved motifs in BAHD acyltransferases, HXXXD and DFGWG, have been implicated in the binding of the acyl-CoA donor and in the structural integrity of the enzyme-donor complex, respectively [5-7]. Our analysis revealed two new motifs, YPLAGR and QVTX(F/L)XCGG, that were conserved across multiple clades. The YPLAGR motif corresponds to the small α-helix-3 (α-3) on the crystal structure of vinorine synthase [42], but no mutagenesis analysis targeting this region has yet been conducted. The lower conservation of this motif in Clade Ia is correlated with a lack of this α-helix and an extra string of 9-14 residues [7], positioned between the corresponding Gly and Arg residues of the YPLAGR motif on the Clade IIIa enzyme vinorine synthase [42]. The QVTX(F/L)XCGG motif is eight amino acids upstream of the HXXXD motif, spanning β-6 and β-7 of vinorine synthase [42], or β-9 and β-10 of Dm3MaT3 [7]. Previous work in vinorine synthase [6] has established the functional importance of the Cys residue in the QVTX(F/L)XCGG motif. A Cys-to-Ala point mutation reduced enzymatic activity by 90%, an impact only exceeded by mutation of the His or Asp residues in HXXXD [6]. Functional support for several clade-specific motifs can also be garnered. The LTFFD motif conserved in Clade Ia maps to α-1 on the Dm3MaT3 crystal structure, near the acyl acceptor binding site [7]. Site-directed mutagenesis of three adjacent α-1 residues on Dm3MaT1 reduced enzyme activity [7], supporting the importance of this motif. Another Clade Ia-specific motif, YFGNC (Additional File 4), is thought to be involved in anthocyanin acyltransferase interactions with malonyl-CoA [7]. Site-directed mutagenesis of vinorine synthase targeting the first Ser residue in the IKPSSPTP motif [6] has also implicated its role in Clade IIIa enzyme function. Given the reported structural diversity of the acyl acceptor binding sites [7], they are more likely to exhibit sequence conservation by clade. In this regard, the suite of clade-specific motifs that we identified should be of value in future structural modelling and mutagenesis studies to understand the diverse enzyme functions in the large BAHD acyltransferase family.

## Conclusions

Our phylogenomic analysis expanded and improved upon the previous BAHD family phylogeny, highlighting two major clades for which almost no biochemical data has yet been generated. Our analysis also identified striking patterns of differential expansion of the BAHD family across five angiosperm taxa, including numerous taxon-specific subclades. This finding may provide a basis for understanding the differentiation of secondary metabolism across taxa. Examining clusters of homologous genes within *Populus *demonstrated that tandem gene duplication has been an important evolutionary force for BAHD diversification within this genus, particularly with respect to two lineage-specific expansions. The retention of salicoid duplicates and likely retrotransposition events have also contributed to the large number of BAHD genes in this taxon. Microarray analysis showed diversity of gene expression among some highly homologous genes in *Populus*, suggesting that some recently duplicated BAHD paralogues have undergone functional divergence in this genus. The discovery of two multi-clade conserved protein motifs as well as clade-specific motifs supports previous research on BAHD enzyme structure and biochemical function, while opening the door to future investigation on the structural basis of donor and substrate specificity within and between clades.

## Methods

### Identification of Putative BAHD family members

Published BAHD acyltransferase sequences [2] were used in initial BLASTP searches against the JGI *Populus trichocarpa *genome v1.1 [23]. Because multiple *in silico *gene prediction programs were used in v1.1, gene models were manually examined for possible structural annotation errors, and to select alternate models if necessary. Gene models located in unanchored short (< 20 kb) scaffolds often represent redundant sequences due to sequence quality or assembly artefacts [25]. When used in BLASTN searches against the *Populus *genome, all putative BAHD sequences from the short scaffolds had high similarity to at least one gene model placed in the 19 linkage groups or larger scaffolds, and were removed from further analysis. The remaining putative BAHD gene models were cross-referenced with the recently released *Populus *genome v2.0 available from the Phytozome website [26], followed again by manual curation (Additional File 1). Manually curated sequences for erroneous gene models are provided in Additional File 9.

Protein sequences of the putative *Populus *BAHD acyltransferases were aligned with previously characterized BAHD proteins ([2] and Additional File 10: SupplementalTable4.xls) using ClustalW [43], then imported into MEGA v.4.0.2 for motif inspection [44]. Sequences which exhibited no HXXXD motif were removed from consideration. Sequences were further screened for a DFGWG-like motif containing at least three of the five amino acids; strict conservation was not required due to known polymorphisms in biochemically characterized BAHD proteins. Exceptions were made for loci highly similar to Clade II members ZmGlossy2 and AtCER, which contain no DFGWG motif. Finally, sequences less than 300 amino acids in length were removed from the list as likely pseudogenes; these sequences either lack the conserved motifs or represent obsolete gene models from the previous genome releases (Additional File 1). BLASTN searches against the NCBI *Populus *EST database revealed no expression support for any of these suspected pseudogenes.

Similar BLASTP searches were conducted against the *Arabidopsis *TAIR9 database [45], the Rice Genome Annotation Database release 5 [46,47], the *Medicago truncatula *genome database MtDB v2.0 [48], and the *Vitis vinifera *genome database (8X) at Genoscope [49]. The sequences were aligned for motif inspection as described above, yielding 55 putative BAHD members in *Arabidopsis*, 84 in *Oryza*, 50 in *Medicago*, and 52 in *Vitis*. For *Oryza*, annotation and final number of genes were determined partially through comparison with the Rice Genome Annotation Database release 6.1. Manual sequence curation revealed two full-length *Arabidopsis *genes previously considered partial sequences [3], and the remaining partial sequences along with several BAHD-like members lacking either of the conserved motifs (Additional File 2) were excluded from our analysis. Protein length was not used as a criterion for further curation in *Medicago*, *Oryza*, or *Vitis*, but unusually long or short models were noted in Additional File 2.

### Phylogenetic Analysis

Putative BAHD protein sequences from *Populus*, *Arabidopsis*, *Oryza*, *Medicago*, and *Vitis *were aligned along with 69 biochemically characterized BAHD members (Additional File 10) using the MAFFT v6.717 online server [50,51]. The FFT-NS-i iterative refinement method was run twice, once with default settings using the BLOSUM62 substitution matrix, and once using the JTT200 substitution matrix. The resulting alignments were imported into BioEdit v7.0.9.0 [52], where any positions containing less than five sequences were designated as gaps and deleted from the alignment. The data were submitted to the CIPRES portal v2.2 [53] for phylogenetic tree construction using RAxML-HPC v7.2.6 [54,55]. Trees were obtained using empirical base frequencies and a maximum likelihood search. The resulting RAxML_bipartitionsBranchLabels.result file was converted to Newick format in Dendroscope v2.3 [56] and imported into MEGA v4.0.2 [44] for visualization. Because the topologies of the maximum likelihood trees resulting from use of the two substitution matrices were broadly consistent with each other, only the BLOSUM62-based tree is shown.

### *In Silico *Characterization of Conserved Protein Motifs

Aligned protein sequences from MAFFT were split into separate FASTA files by clade using BioEdit, and subjected to motif analysis using the MINER v2.0 web interface with default settings [16-18]. Because a minimum of 25 sequences is recommended by the program to achieve good statistical support, Clade IV (five members) and Clade II (18 members) were excluded from the analysis. Putative motifs were identified based on a phylogenetic similarity z-score threshold automatically determined by the MINER. Previous work suggests thresholds of -1.5 to -2.2 are typical [18]; actual thresholds in our study ranged from -2.05 to -2.28. The corresponding sequence alignments for multi-clade motifs were manually trimmed to 25 amino acids, including bordering residues, and submitted to Weblogo v2.8.2 [57] for visualization. Sequence alignments corresponding to representative clade-specific motifs were also trimmed to 10 amino acids and submitted to Weblogo for visualization. Any motifs lacking at least one amino acid conserved at a rate >1.5 bits were not reported.

Putative subcellular localization for all BAHD proteins by clade was examined using WoLF PSORT [58,59], Predotar [60], and TargetP [61,62], assigning "plant" as the organism type. The predicted subcellular localization site (mitochondrial, chloroplast, secretory organelles, or any others) for each protein was noted, and overall patterns were summarized for each clade.

### Visualization of Putative BAHD Genes on *Populus *Linkage Groups and Identification of Gene Duplication Events

The chromosomal locations of the 100 *Populus *BAHD genes were visualized in ideograms using the software package from Böhringer et al. [63], based on the *Populus trichocarpa *genome v2.0. Syntenous segments of the genome derived from the "salicoid" genome-wide duplication event [23] were color-coded according to the position information provided in the SalicaceaeDup.seg file downloaded from Phytozome [26]. Two types of duplication events were noted: genome-wide duplications originating from the salicoid event, and local duplications. Salicoid duplications were identified according to Tuskan et al. [23] based on the SalicaceaeDup.ort.txt file from Phytozome [26]. Because many of the *in silico *gene model predictions have not been validated (*e.g*., some represent partial gene models or transposons), the "local duplications" category is used here to include tandem or tandem array duplications with no intervening predicted gene models (Additional File 6). Neither partial BAHD acyltransferase sequences nor transposons were counted as intervening gene models. Three cases deserve special mention. One appears to be a two-gene tandem duplication, involving POPTR_0011s12480 + POPTR_0011s12490 (AATL16) and POPTR_0011s12500 + POPTR_0011s12510 (AATL17). AATL16 and 17 were therefore retained as a local duplication pair in our analysis. Another involves AATL12-13 vs. AATL14 with an intervening partial BAHD gene model (POPTR_0010s06400). AATL14 is a salicoid duplicate of AATL11, and shares less than 40% protein sequence similarity with the highly homologous AATL12 and AATL13 (98% similarity). AATL14 was thus excluded as part of the tandem array. The other case involves a six-gene tandem array (HMTL1-6), separated by a non-BAHD gene model POPTR_0001s45170. Several discrepancies were noted for this region between the two genome assembly versions. The intervening gene model prediction corresponded to a full-length disease resistance protein in v1.1 (eugene3.00012870) but to a partial one in v2.0 (POPTR_0001s45170). HMTL3 was predicted in an opposite orientation relative to other genes within this region in v2.0, but the corresponding HMTL2 (eugene3.0012871), HMTL3 (eugene3.0012869) and the intervening gene models in v1.1 were in the same orientation. The predicted tandem copies also varied between the two versions, presumably due to the difficulty in assembling highly similar sequences. For all these reasons, we tentatively assigned HMTL1-2 and HTML3-6 (including the inverted HMTL3 locus) to two separate tandem duplication blocks in our analysis (Additional File 6).

To search for retrotransposons, BioPerl SeqIO was used to extract the 10-kb sequences immediately upstream and downstream of each of the 100 putative *Populus *BAHD acyltransferases from the v2.0 genome. Sequences were subjected to BLASTX searches against the GenBank non-redundant protein database with an *E*-value cutoff of 1e^-10^. The output file was processed with the BioPerl SearchIO scripts, and the results were manually inspected to determine whether the regions of interest were likely to contain retrotransposons based on the descriptions of matches. Only sequences with multiple hits to retrotransposon elements were documented (Additional File 6).

### Microarray Data Mining

Affymetrix *Populus *microarray datasets generated in our laboratory [24] were used to investigate BAHD gene expression across genotypes, tissues, and stress treatments. These arrays corresponded to nine experimental groups, including 1) nitrogen-stressed young and expanding leaves of two *Populus fremontii *× *angustifolia *genotypes (1979 and 3200), 2) systemic young and expanding leaves of *Populus fremontii *× *angustifolia *genotype RM5 one week after lower leaf wounding, or systemic expanding leaves and root tips 90 h post-wounding, 3) expanding leaves of *P. tremuloides *genotype 271 following detopping, and 4) methyl jasmonate-elicited suspension cell cultures of *P. tremuloides *genotype L4. All experiments contained respective non-stressed controls and two biological replicates. The arrays were pre-processed by the GC-RMA algorithm using GeneSpring GX 11.0.2 (Agilent Technologies Inc.). *Populus *probes exhibiting mean raw hybridization intensities of at least 50 in any experimental group were flagged as "present", yielding a list of 24,871 probes, and the rest designated as "absent" and excluded from analysis. Hierarchical clustering was performed using several distance metrics to evaluate the sample clustering patterns. All control and treatment samples from the same experimental group clustered together, except for the expanding leaves from the one week wounding experiment. These arrays were excluded from further analysis. Based on the POParray database [25] and the v2.0 poplar genome [26], the filtered list contained a total of 60 probes annotated as BAHD acyltransferases, representing 48 unique BAHD genes. Because the Affymetrix array was designed based on the v1.0 genome release and a large collection of ESTs from several *Populus *species, redundancy is a known issue [25]. To minimize redundant representation, we further reduced the list of 60 probes to those that have unique gene matches, and in cases of multi-probe representation, to those that exhibited the highest hybridization signals consistently across multiple samples. The final list included 36 probes with unique gene representation, and 5 probes matching to multiple highly similar genes. The list of BAHD acyltransferase gene-to-probe correspondences can be found in Additional File 11: SupplementalTable5.xls. The BAHD probe expression values from all control samples across genotypes and tissues were grouped by clade and log_10_-transformed for visualization using the Heatmapper *Plus *tool at the Bio-Array Resource for Plant Functional Genomics [64,65]. Stress responses of BAHD genes were also visualized in heatmaps using log_2_-transformed expression ratios of experimental treatments relative to control samples.

### Gene Expression Correlation Analysis

Log-transformed microarray data was imported in to JMP v8.0 (SAS Institute, Inc.) and distribution of expression values for each gene probe was analyzed using histogram plots. The majority of probes did not generate curves similar to a normal distribution. Therefore, we used Spearman's ρ as a non-parametric measure of pairwise correlation for gene expression among genes within each clade. We then organized gene pairs by duplication type (local, salicoid or other) according to Additional File 6 generating box plots for each using SigmaStat v3.5 (Systat Software Inc). For the salicoid duplicates that have also been associated with more recent local duplications, all possible pairwise comparisons between the lone salicoid member and the local duplicates (e.g., CHATL6 vs. CHATL1-3, and HMTL7 vs. HMTL1-6) were included. Kruskall-Wallis one-way ANOVA on Ranks was used to test for differences among any duplication categories, followed by a *post-hoc *Dunn's Method test for pairwise differences between categories.

### Quantitative Real Time RT-PCR Analysis

Apices, leaves at leaf plastochron index (LPI) 0-1 and LPI 8, internodes corresponding to LPI 1-4 and LPI 7-10, and root tips of *P. tremuloides *genotype 271 were flash frozen and ground under liquid nitrogen for RNA extraction. Male and female flowers were collected from wild *P. tremuloides *at field sites near Houghton, Michigan. RNA was extracted from three biological replicates of all samples using the CTAB method [66], quantified via Nanodrop spectrophotometry and quality-checked on a 1% agarose gel. cDNA was synthesized with 5.0 μg of RNA using dT20-VN primers and SuperScript II reverse transcriptase (Invitrogen). RNA samples from the nitrogen stress microarray experiments detailed above were also used to generate cDNA samples with two biological replicates per condition.

QPCR reactions were carried out in a 12.5 μl reaction volume using cDNA equivalent to 2.5 ng of total RNA, 100 nM each of forward and reverse primers, and the ABsolute™ SYBR Green Master Mix (ABgene) with 0.003% ROX reference dye. Two technical replicates were included for each sample, and sample plates were run on the Mx3005P™ (Stratagene). Relative expression was calculated by the ΔCt method using the geometric mean of three housekeeping genes (elongation factor β1, cyclophilin, and ubiquitin-conjugating enzyme E2), except for the nitrogen experiment where the last housekeeping gene was excluded due to missing data for some samples. PCR amplification efficiency was calculated using the LinRegPCR program [67]. Primers were designed based on the predicted transcript sequences of the target *P. trichocarpa *gene models and the corresponding GenBank *Populus *ESTs, and wobbles were introduced wherever variation exists. The primer sequences are: CHATL1/2 forward AGTTWCWTGCAGACACCGAGCGTA, and reverse AGGGCAATGGYMCGACATATCCAA; CHATL3/6 forward TGGCCCTTCAGARATRTCTGCTCT, and reverse AGTCACGTCAGCCTTRGCCTTTCT; CHATL4/5 forward ACACCACTGACAACGTTCCGCTTA, and reverse TGTTGCCATTGCCACTGAGTATGC; elongation factor 1β forward AAGAGGACAAGAAGGCAGCA, and reverse CTAACCGCCTTCTCCAACAC; cyclophilin forward ATGGCTTGATGGGAAACAT, and reverse AATCTCATTAGGATCATTAAAGGACAG; and ubiquitin-conjugating enzyme E2 forward CTGAAGAAGGAGATGACARCMCCA, and reverse GCATCCCTTCAACACAGTTTCAMG.

## Authors' contributions

LKT conducted BAHD sequence alignment and manual annotation, performed phylogenetic analysis, motif identification, microarray data analysis, QPCR, and drafted the manuscript. VEJ conducted all BLAST searches, handled large-scale data extraction from external databases, and developed ideograms. CJT conceived of and coordinated the study, participated in BAHD annotation and microarray data analysis, and revised the manuscript. All authors read and approved the final manuscript.

## Supplementary Material

Additional file 1 **Summary of Putative BAHD Acyltransferases in the *Populus trichocarpa *Genome**. BAHD acyltransferase loci are listed by clade, then by corresponding JGI v2.0 and v1.1 gene models and previously assigned names [3]. Protein length, exon number, and intron number are included along with manual curation notes. Click here for file

Additional file 2 **Summary of Putative BAHD Acyltransferases in *Arabidopsis thaliana*, *Medicago truncatula*, *Oryza sativa*, and *Vitis vinifera *Genomes**. BAHD acyltransferase loci are listed alphabetically by genus, then by clade, then by locus number. Click here for file

Additional file 3 **Detailed Views of Phylogenetic Relationships Within Clades Ib, II, IIIb, IV, and Vb**. Coloration of clades and symbols are as described in Figures 1, 2, 3, 4. Click here for file

Additional file 4 **Additional Clade-Specific Motifs Identified by MINER**. Motifs are arranged by clade, and bordered with the same color scheme as in Figure 1. The thickly boxed motif in Clade Ia overlaps with the range for the QVTX(F/L)XCGG motif shown in Figure 5. Clade Ib had no additional motifs beyond those shown in Figure 5. Click here for file

Additional file 5 **Analysis of BAHD Acyltransferase Protein Subcellular Localization**. Each chart indicates the results from a different prediction algorithm, with the number of sequences indicated by the y-axis and clade indicated on the x-axis Click here for file

Additional file 6 **Duplications and Retrotransposons Associated With *Populus *BAHD Acyltransferase Genes**. Genes are organized as in Additional file 1. Click here for file

Additional file 7 **Pairwise Gene Expression Correlation Across *Populus *BAHD Acyltransferase Duplication Types**. Box plots for Spearman rank correlations of pairwise gene expression by clade across all microarray experiments. Gene pairs are grouped by their association with local duplication, salicoid duplication, or others (all other pairwise combinations). Categories with the same letter had median correlation values that were not significantly different at α = 0.05 according to Dunn's Multiple Comparison test. Click here for file

Additional file 8 **QPCR Expression Analysis of *Populus CHATL *Genes**. A: Relative expression of the highly similar *CHATL1/2*, *CHATL3/6 *and *CHATL4/*5 gene pairs in various *P. tremuloides *tissues. Data represent means ± SE of three biological replicates. Tissues examined included apical bud/leaves (Apex), young leaves (LPI 0/1), mature leaves (LPI 8), internodes 1-4 (IN 1-4) and 7-10 (IN 7-10), root tips (Root), female flowers (F Flwr), and male flowers (M Flwr). Dashed orange line indicates an expression level comparable to the presence vs. absence cutoff used in microarray analysis. B: Relative expression of *CHATL *genes in young (YL) and expanding (EL) leaves from the nitrogen stress experiment. Data represent means ± SD of two biological replicates. Genotypes are listed as in Figure 7A, with "High N" samples corresponding to non-stressed tissues in Figure 7A. Click here for file

Additional file 9 **Manually Curated *Populus *BAHD Acyltransferase Protein and CDS Sequences**. Data provided for sequences noted in Additional File 1. Click here for file

Additional file 10 **Biochemically Characterized BAHD Acyltransferases Included in the Phylogenetic Analysis**. All biochemically characterized BAHD proteins included in our analysis are listed by clade and by their order of appearance (from top to bottom) in the detailed phylogenies Click here for file

Additional file 11 **Correspondences of *Populus *BAHD Acyltransferase Genes and Affymetrix Probe Identifiers**. Coloration for gene name is assigned according to clade membership in Figure 1. Click here for file
