# Supplementary figures and images for: Differential phylogenetic expansions in BAHD acyltransferases across five angiosperm taxa and evidence of divergent expression among Populus paralogues
Source: BMC Genomics. 2011 May 12;12:236. doi: 10.1186/1471-2164-12-236 (PMC3123328; doi:10.1186/1471-2164-12-236)

## Clade Ib

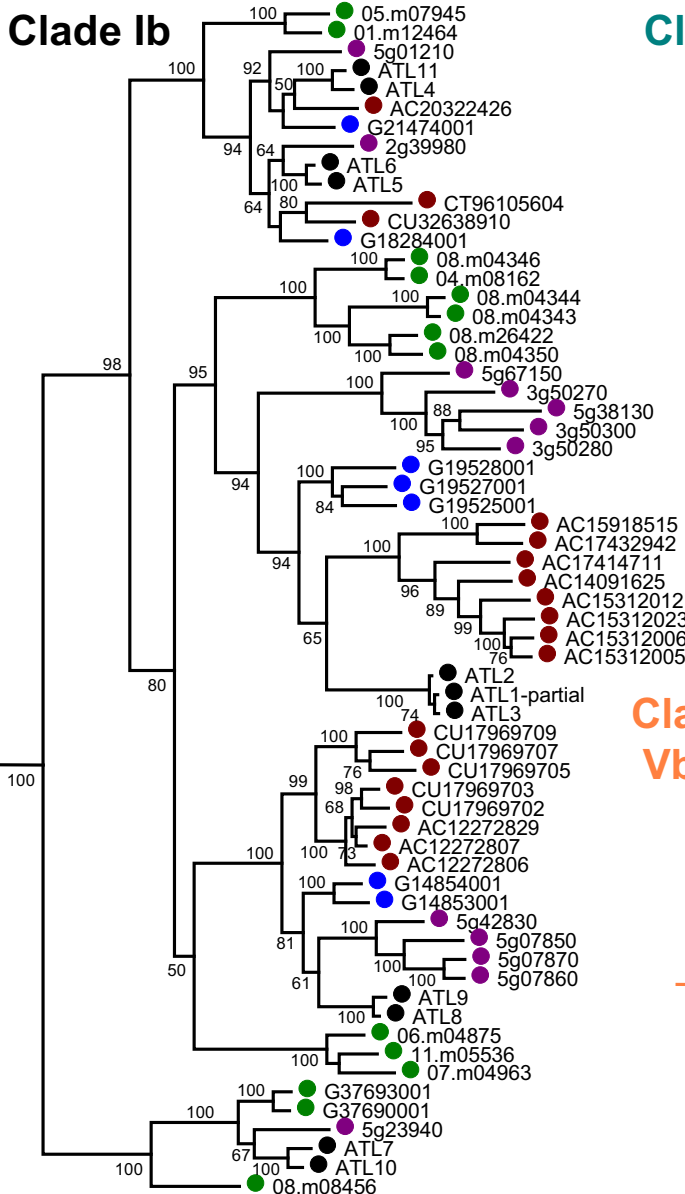

## Clade IIIb

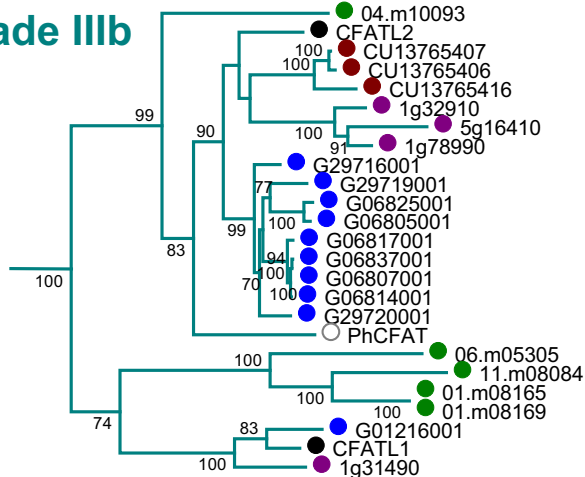

## Clade IV

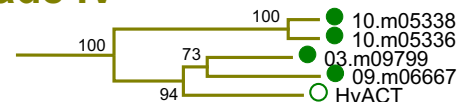

## Clade Vb

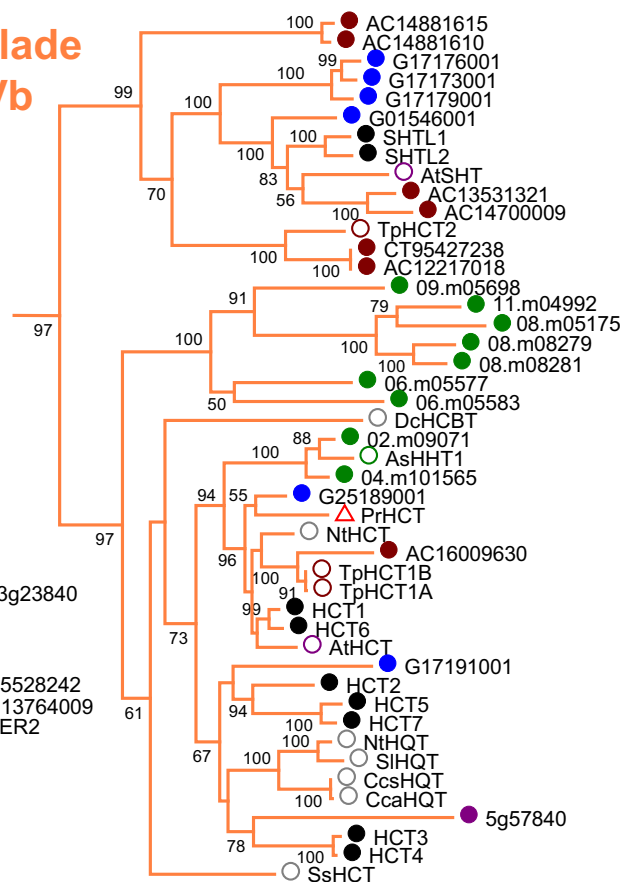

## Clade II

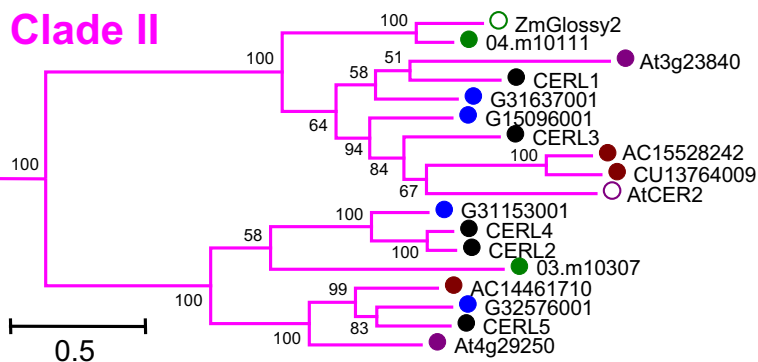

0.5

Supplement: Additional file 3 — Detailed Views of Phylogenetic Relationships Within Clades Ib, II, IIIb, IV, and Vb. Coloration of clades and symbols are as described in Figures 1, 2, 3, 4. [file 1471-2164-12-236-S3.PDF]

Ia

IIIa

IIIb

Va

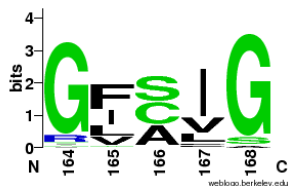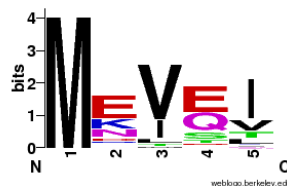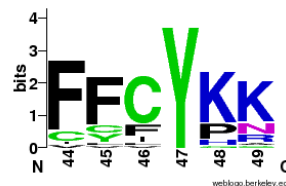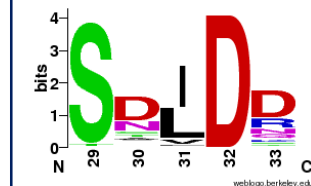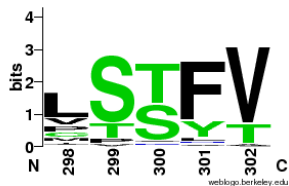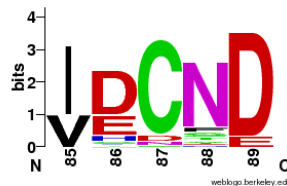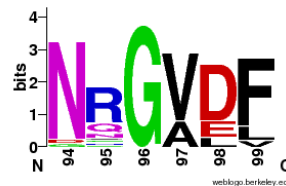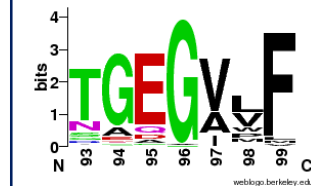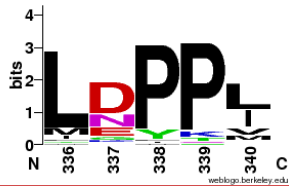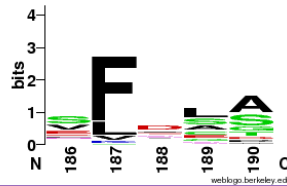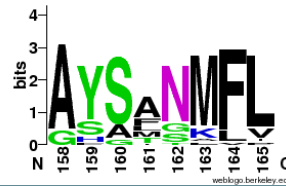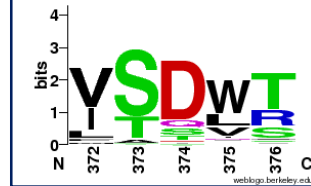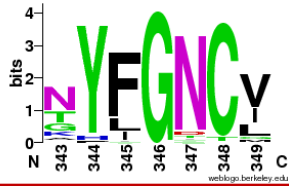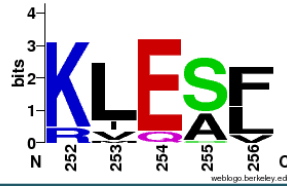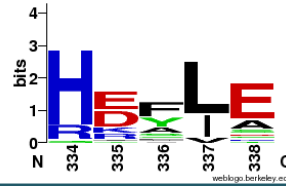

Vb

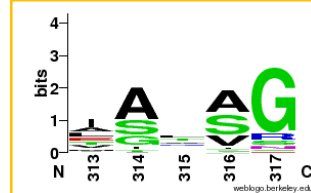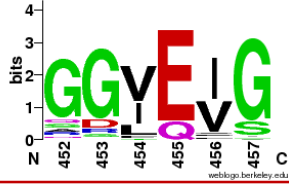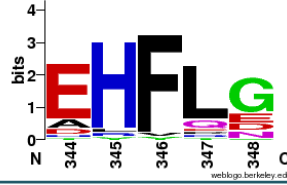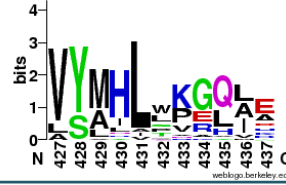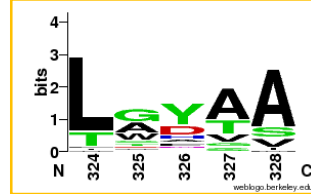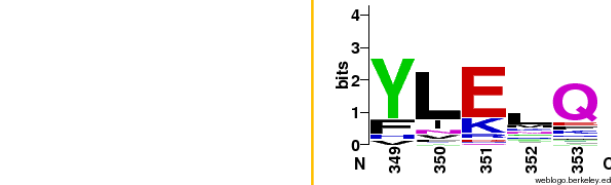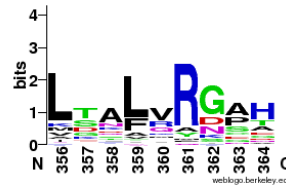

Additional Clade-Specific Motifs Identified by MINER

Supplement: Additional file 4 — Additional Clade-Specific Motifs Identified by MINER. Motifs are arranged by clade, and bordered with the same color scheme as in Figure 1. The thickly boxed motif in Clade Ia overlaps with the range for the QVTX(F/L)XCGG motif shown in Figure 5. Clade Ib had no additional motifs beyond those shown in Figure 5. [file 1471-2164-12-236-S4.PDF]

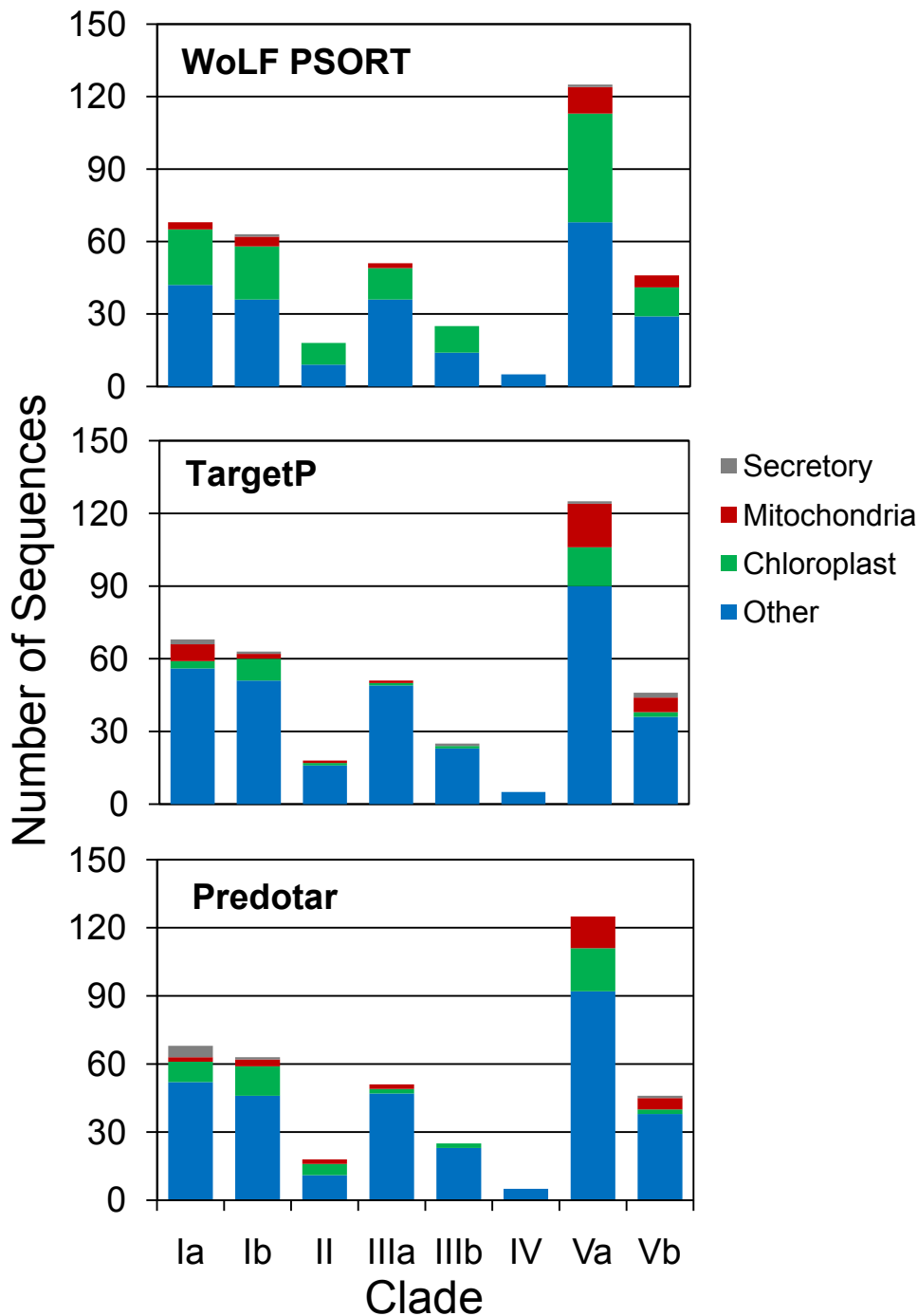

## Analysis of BAHD Acyltransferase Protein Subcellular Localization

Supplement: Additional file 5 — Analysis of BAHD Acyltransferase Protein Subcellular Localization. Each chart indicates the results from a different prediction algorithm, with the number of sequences indicated by the y-axis and clade indicated on the x-axis [file 1471-2164-12-236-S5.PDF]

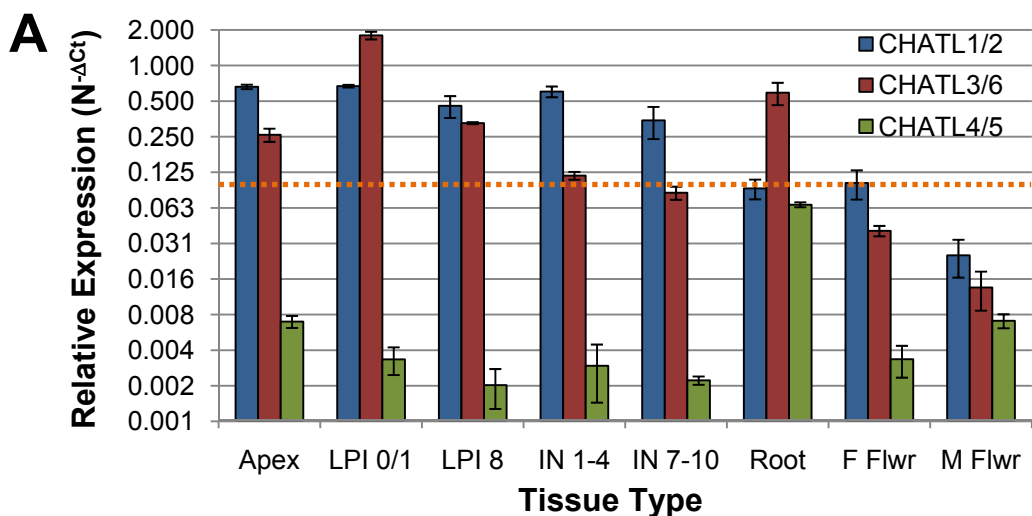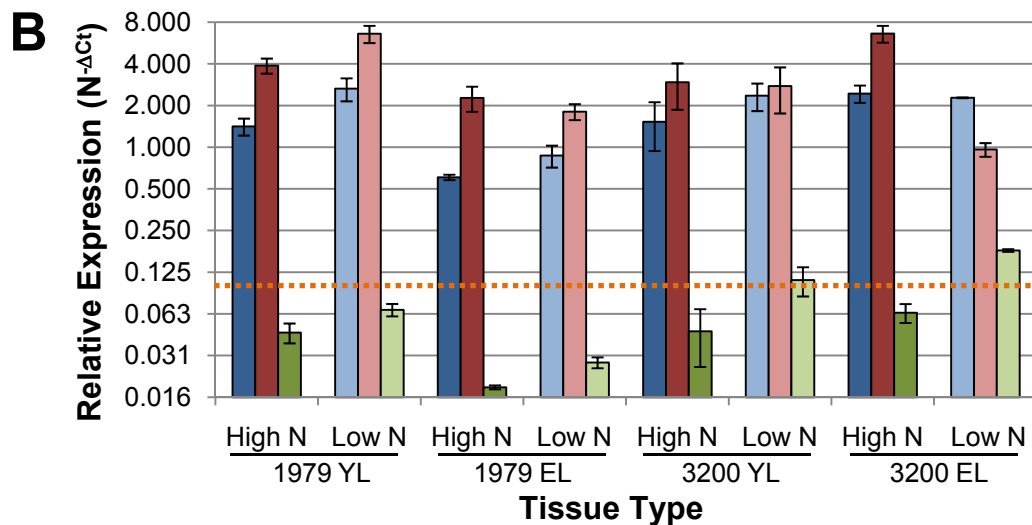

# QPCR Expression Analysis of *Populus* CHATL Genes

Supplement: Additional file 8 — QPCR Expression Analysis of Populus CHATL Genes. A: Relative expression of the highly similar CHATL1/2, CHATL3/6 and CHATL4/5 gene pairs in various P. tremuloides tissues. Data represent means ± SE of three biological replicates. Tissues examined included apical bud/leaves (Apex), young leaves (LPI 0/1), mature leaves (LPI 8), internodes 1-4 (IN 1-4) and 7-10 (IN 7-10), root tips (Root), female flowers (F Flwr), and male flowers (M Flwr). Dashed orange line indicates an expression level comparable to the presence vs. absence cutoff used in microarray analysis. B: Relative expression of CHATL genes in young (YL) and expanding (EL) leaves from the nitrogen stress experiment. Data represent means ± SD of two biological replicates. Genotypes are listed as in Figure 7A, with "High N" samples corresponding to non-stressed tissues in Figure 7A. [file 1471-2164-12-236-S8.PDF]
