# Supplementary material for: Differential phylogenetic expansions in BAHD acyltransferases across five angiosperm taxa and evidence of divergent expression among Populus paralogues
Source: BMC Genomics. 2011 May 12;12:236. doi: 10.1186/1471-2164-12-236 (PMC3123328; doi:10.1186/1471-2164-12-236)
Supplement: Additional file 7 — Pairwise Gene Expression Correlation Across Populus BAHD Acyltransferase Duplication Types. Box plots for Spearman rank correlations of pairwise gene expression by clade across all microarray experiments. Gene pairs are grouped by their association with local duplication, salicoid duplication, or others (all other pairwise combinations). Categories with the same letter had median correlation values that were not significantly different at α = 0.05 according to Dunn's Multiple Comparison test. [file 1471-2164-12-236-S7.PDF]

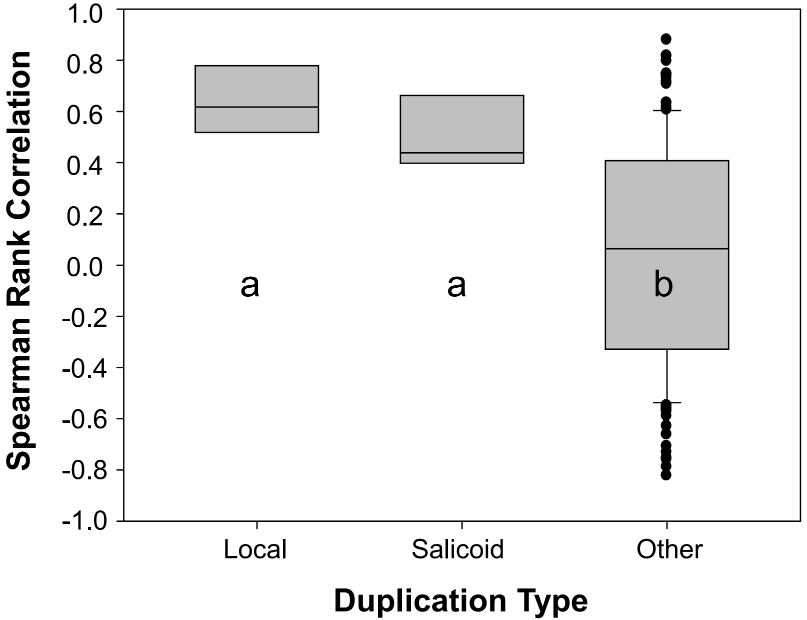

Pairwise Gene Expression Correlation Across *Populus* BAHD Acyltransferase Duplication Types
